# Deletion of Cytoplasmic Double-Stranded RNA Sensors Does Not Uncover Viral Small Interfering RNA Production in Human Cells

**DOI:** 10.1128/mSphere.00333-17

**Published:** 2017-08-16

**Authors:** Susan Schuster, Lotte E. Tholen, Gijs J. Overheul, Frank J. M. van Kuppeveld, Ronald P. van Rij

**Affiliations:** a Department of Medical Microbiology, Radboud University Medical Center, Radboud Institute for Molecular Life Sciences, Nijmegen, The Netherlands; b Virology Division, Department of Infectious Diseases and Immunology, Faculty of Veterinary Medicine, Utrecht University, Utrecht, The Netherlands; University of Chicago

**Keywords:** RNA interference, RNA virus, innate immunity

## Abstract

Antiviral immunity in insects and plants is mediated by the RNA interference (RNAi) pathway in which viral long double-stranded RNA (dsRNA) is processed into small interfering RNAs (siRNAs) by Dicer enzymes. Although this pathway is evolutionarily conserved, its involvement in antiviral defense in mammals is the subject of debate. In vertebrates, recognition of viral RNA induces a sophisticated type I interferon (IFN)-based immune response, and it has been proposed that this response masks or inhibits antiviral RNAi. To test this hypothesis, we analyzed viral small RNA production in differentiated cells deficient in the cytoplasmic RNA sensors RIG-I and MDA5. We did not detect 22-nucleotide (nt) viral siRNAs upon infection with three different positive-sense RNA viruses. Our data suggest that the depletion of cytoplasmic RIG-I-like sensors is not sufficient to uncover viral siRNAs in differentiated cells.

**IMPORTANCE** The contribution of the RNA interference (RNAi) pathway in antiviral immunity in vertebrates has been widely debated. It has been proposed that RNAi possesses antiviral activity in mammalian systems but that its antiviral effect is masked by the potent antiviral interferon response in differentiated mammalian cells. In this study, we show that inactivation of the interferon response is not sufficient to uncover antiviral activity of RNAi in human epithelial cells infected with three wild-type positive-sense RNA viruses.

## Abstract (precis)

The contribution of the RNA interference (RNAi) pathway in antiviral immunity in vertebrates has been widely debated. It has been proposed that RNAi possesses antiviral activity in mammalian systems but that its antiviral effect is masked by the potent antiviral interferon response in differentiated mammalian cells. In this study, we show that inactivation of the interferon response is not sufficient to uncover antiviral activity of RNAi in human epithelial cells infected with three wild-type positive-sense RNA viruses.

## OBSERVATION

The RNA interference (RNAi) pathway is the primary antiviral pathway in insects, nematodes, plants, and fungi, whereas mammals have evolved sophisticated type I interferon (IFN)-based innate and adaptive immune responses to combat viruses. The hallmark of an antiviral RNAi response in insects is the processing of viral double-stranded (dsRNA) into 21-nucleotide (nt) viral small interfering RNAs (vsiRNAs) by the RNase III enzyme Dicer-2 and its cofactor R2D2 (1, 2). These vsiRNAs are loaded into the Argonaute 2-containing RNA-induced silencing complex (RISC), after which one of the strands (passenger) is eliminated and the other strand (guide) is retained to guide the recognition and cleavage of complementary viral RNAs (1, 2).

In differentiated mammalian cells, viral pathogen-associated molecular patterns are sensed by pattern recognition receptors to induce IFN expression. For example, viral dsRNA and other nonself RNA signatures are recognized by the DEAD box helicases RIG-I (retinoic acid-inducible gene I) and MDA5 (melanoma differentiation-associated protein 5) (3). Sensing of dsRNA by these RIG-I-like receptors activates a signaling cascade that leads to transcriptional induction of type I IFN genes and ultimately to an antiviral and proinflammatory response (3). IFN activates autocrine and paracrine signaling through the JAK/STAT pathway, resulting in the production of hundreds of interferon-stimulated genes (ISGs), which possess a wide range of antiviral activities, including the degradation of nucleic acids by the 2′,5′-oligoadenylate synthetase (OAS)–RNase L system (4, 5).

It is hotly debated whether RNAi contributes to antiviral defense in mammals (6–13). Although several studies found little accumulation of vsiRNAs upon infection of somatic cells with different viruses (14–16), recent publications suggest that this evolutionarily conserved pathway possesses antiviral activity under specific experimental conditions (7–9). Several hypotheses have been proposed about the specific conditions under which antiviral RNAi is detectable in mammals. First, vsiRNAs of ∼22 nt, the expected length of mammalian Dicer products, were identified in several mammalian cell types and suckling mice infected with viruses lacking their viral suppressor of RNAi (VSR) (7, 8, 17). In these studies, vsiRNAs were not detectable when wild-type viruses were used, suggesting that VSRs efficiently suppress vsiRNA production. Second, vsiRNAs could also be detected in undifferentiated mouse embryonic stem cells (mESCs) infected with wild-type encephalomyocarditis virus (EMCV) (9). Interestingly, vsiRNA production was lost after differentiation of mESCs into an ectodermal cell lineage, suggesting that certain features associated with stemness allow recognition of viral RNA by the RNAi machinery (9). One of these features could be the absence of an efficient innate antiviral immune response. mESCs do not produce type I interferons (IFNs) and express lower levels of ISGs than fibroblasts upon viral infection and stimulation with dsRNA mimics (18, 19). Similarly, it was shown that human ESCs possess an attenuated cytoplasmic dsRNA response and fail to induce IFN-β expression (20).

A third hypothesis to explain the absence or presence of antiviral RNAi is that the IFN response masks or inhibits RNAi in differentiated mammalian cells (13). Similarly, as RIG-I and MDA5 have been shown to stably bind to viral RNA (21, 22), physical competition between Dicer and RIG-I-like receptors for dsRNA may occur. Indeed, a recent study found that RNAi was efficient in MAVS- and IFNAR1-deficient cells upon treatment with synthetic dsRNA (13), but it had not been directly tested whether genetic inactivation of the interferon response results in production of vsiRNAs.

In this study, we tested the hypothesis that the type I IFN response competes with RNAi-dependent processing of viral dsRNA. To this end, we analyzed vsiRNA production of three positive-sense RNA viruses in cells lacking both of the cytosolic RNA sensors RIG-I and MDA5. For our study, we chose wild-type viruses from three distinct virus families, Sindbis virus (SINV; *Togaviridae*), yellow fever virus 17D vaccine strain (YFV17D; *Flaviviridae*), coxsackievirus B3 (CBV3; *Picornaviridae*). SINV and YFV are arthropod-borne viruses for which it has been shown that viral dsRNA is efficiently processed into vsiRNAs in their mosquito vector (2). For another *Picornaviridae* family member, EMCV, vsiRNA production has been demonstrated in mESCs. Although previous studies found that inactivation or deletion of VSRs renders viruses sensitive to antiviral RNAi (7–9), we specifically chose to analyze wild-type viruses here, to directly assess whether inactivation of the IFN response is sufficient to uncover vsiRNA production. Although a VSR has been proposed in one of the viruses used (YFV [23]), no VSR has been identified to date in SINV and CBV3 (see Text S1 in the supplemental material). Moreover, using luciferase-based RNAi reporter assays, we were unable to detect RNAi-suppressive activities in HeLa cells infected with SINV, YFV17D, and CBV3 (see Fig. S1 in the supplemental material).

10.1128/mSphere.00333-17.5 TEXT S1  Supplemental results and materials and methods. Download TEXT S1, PDF file, 0.2 MB. Copyright © 2017 Schuster et al. 2017 Schuster et al. This content is distributed under the terms of the Creative Commons Attribution 4.0 International license.

10.1128/mSphere.00333-17.1 FIG S1  No evidence for RNAi-suppressive activity in SINV, YF17D, and CBV3 infection. (A) Argonaute 2 dependency of luciferase-based RNAi reporter assay in mammalian cells. Wild-type (+/+) and Argonaute 2 mutant (−/−) mouse embryonic fibroblasts (MEFs) were transfected with expression plasmids encoding firefly (Luc) and renilla (Ren) luciferase along with a plasmid carrying a short hairpin RNA (shRNA) targeting Luc or a nontargeting control shRNA. Luc counts were normalized using Ren luciferase counts and expressed relative to control shRNA. (B) RNAi reporter assay in HeLa cells infected with SINV (MOI of 0.1) for 24 h, YFV17D (MOI of 0.1) for 46 h, or CBV3 (MOI of 0.01) for 8 h or in mock-infected cells (24 h). (C) RNAi reporter assay in noninfected and SINV-infected *Drosophila* S2 cells. S2 cells were infected with a Sindbis virus recombinant expressing the blasticidin resistance gene, and infected cells were selected under blasticidin selection and used in an RNAi reporter assay along with noninfected S2 cells. Two independently selected populations of SINV-infected cells were used (clones 1 and 2). To assess RNAi efficiency, cells were transfected with expression plasmids encoding Luc and Ren luciferase along with dsRNA targeting Luc or nontargeting control dsRNA (green fluorescent protein [GFP]). Data are presented as means and standard deviations (SD) from 3 biological replicates. ns, nonsignificant (Student’s *t* test). Download FIG S1, PDF file, 0.8 MB. Copyright © 2017 Schuster et al. 2017 Schuster et al. This content is distributed under the terms of the Creative Commons Attribution 4.0 International license.

**FIG 1:**
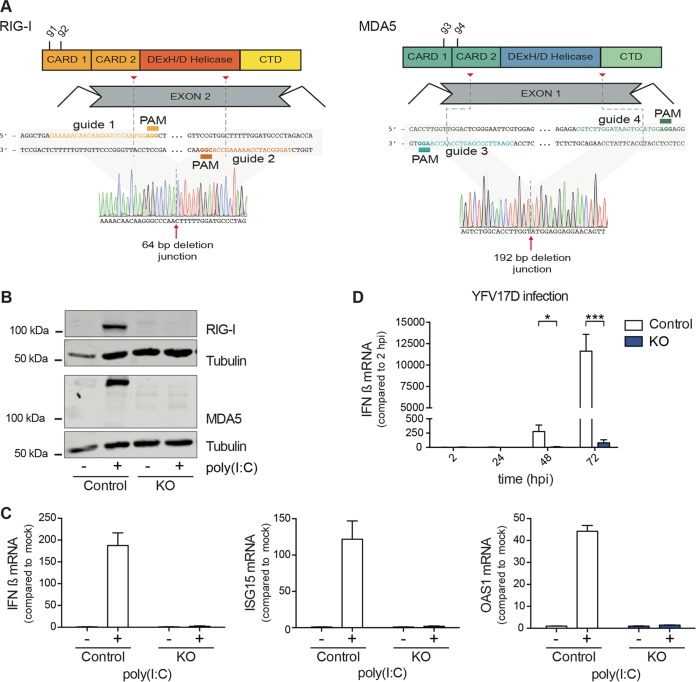
Functional validation of RIG-I- and MDA5-deficient cell line generated by CRISPR-Cas9 technology. (A) Schematic representation of the RIG-I and MDA5 domain structure, the guide RNAs, and the Sanger sequence obtained. CTD, C-terminal domain. (B) Western blotting for RIG-I, MDA5, and tubulin as a loading control. Control and RIG-I- and MDA5-deficient (KO) cells were mock treated or stimulated with 200 ng poly(I⋅C) for 24 h. See Fig. S1 in the supplemental material for the complete image. (C) Control and KO cells were treated with 200 ng of poly(I⋅C) or mock treated for 24 h, and the induction of IFN-β, ISG15, and OAS1 mRNA was measured by RT-qPCR. (D) Control and KO cells were infected with yellow fever virus 17D (YFV17D) at an MOI of 0.1, and the induction of IFN-β mRNA was measured by RT-qPCR over time (hpi, hours postinfection). Data are presented as means and standard deviations from biological triplicates. One representative experiment of three independent repeats is shown. Statistical significance was determined using Student’s *t* test (***, *P* < 0.001; **, *P* < 0.01; *, *P* < 0.05).

We used clustered regularly interspaced short palindromic repeat (CRISPR)-Cas9 technology to generate a HeLa clonal cell line that is deficient in both RIG-I and MDA5. Sanger sequencing confirmed a 64-bp out-of-frame deletion in exon 2 in the RIG-I gene (Fig. 1A). For MDA5, CRISPR-Cas9 induced cleavage around the predicted site in exon 1, resulting in an in-frame deletion of 64 amino acids in the CARD domains (Fig. 1A). Western blot analysis confirmed the absence of detectable RIG-I and MDA protein upon poly(I⋅C) stimulation for the RIG-I–MDA5 knockout (KO) cell line (Fig. 1B and S2).

10.1128/mSphere.00333-17.2 FIG S2  Western blot assay for RIG-I, MDA5, and tubulin. The control and RIG-I- and MDA5-deficient (KO) cells were mock treated or stimulated with 200 ng poly(I⋅C) for 24 h. This is the full image of the Western blot shown in Fig. 1B. Download FIG S2, PDF file, 2.1 MB. Copyright © 2017 Schuster et al. 2017 Schuster et al. This content is distributed under the terms of the Creative Commons Attribution 4.0 International license.

To validate that the KO cell line was deficient in RIG-I and MDA5 activity, we stimulated the cells with the dsRNA mimic poly(I⋅C) and measured IFN-β, ISG15, and OAS1 mRNA expression by reverse transcription-quantitative PCR (RT-qPCR) (Fig. 1C). Upon poly(I⋅C) stimulation, the RIG-I- and MDA5-deficient cells expressed significantly lower levels of IFN-β, ISG15, and OAS1 than the control cells, confirming that these cell lines are functionally deficient in dsRNA sensing (Fig. 1C). To confirm these data, we infected KO and control cells with YFV17D, infection of which is sensed by both RIG-I and MDA5 (24). Indeed, we found that IFN-β induction was strongly reduced in KO cells at all time points (Fig. 1D). Taken together, these results indicate that the RIG-I–MDA5 cell line is deficient in RNA sensing upon virus infection and direct stimulation with dsRNA.

**FIG 2:**
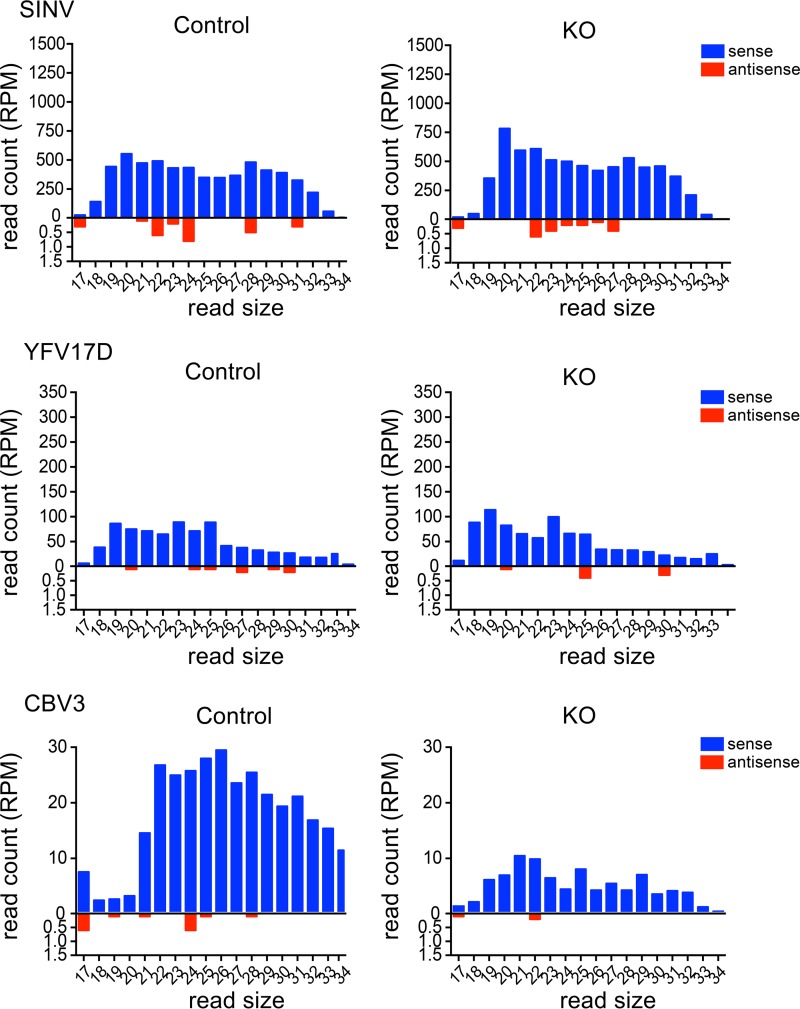
Size profile of viral small RNAs obtained from control cells and RIG-I- and MDA5-deficient (KO) cells after infection for 24 h with SINV at an MOI of 0.1, for 48 h with YFV17D at an MOI of 0.1, or for 16 h with CBV3 at an MOI of 0.01. Small RNAs were mapped to the sense (blue bars) and antisense (red bars) strands of the viral genome, allowing 1 mismatch. Sense and antisense reads are presented on different scales, as antisense reads were very scarce. RPM, reads per million.

We next investigated whether an antiviral RNAi response is induced in an interferon-deficient background by analyzing small RNAs by deep sequencing. Specifically, processing of viral dsRNA by human Dicer is expected to produce vsiRNA of 22 nt in size (7–9). We first established the replication kinetics for SINV, YFV17D, and CBV3 in control and KO cells (Fig. S3) to select time points at which the viruses replicate to high levels but do not yet cause extensive cell death. We next generated deep-sequencing libraries of size-selected RNA (19 to 30 nt) from control HeLa cells and the RIG-I- and MDA5-deficient KO cells infected with SINV, YFV17D, or CBV3. As expected, the majority of unmapped reads of all libraries ranged in size from 21 to 24 nt (Fig. S4), with a clear peak at 22 nt. As expected, the 22-nt fraction consists mainly of cellular microRNAs (miRNAs), as 89 to 94% of the reads mapped to human miRNAs. In contrast, virus-derived small RNAs revealed a broad distribution of reads, ranging in size from 17 to 31 nt for all viruses, without enrichment for 22-nt reads (Fig. 2). Moreover, viral small RNA reads were strongly biased for reads mapping to the sense strand of the viral genome, unlike Dicer-dependent vsiRNAs of RNA viruses in insects and mammals, which map to the viral sense and antisense strands in similar numbers (2, 7, 9, 17).

10.1128/mSphere.00333-17.3 FIG S3  Replication kinetics of SINV, YFV17D, and CBV3. Control and RIG-I- and MDA5-deficient (KO) cells were infected with SINV and YFV17D at an MOI of 0.1 and CBV3 at an MOI of 0.01, and viral titers (50% tissue culture infective dose [TCID_50_]/ml) were determined at different time points. Data represent means and standard deviations from three biological replicates. Statistical significance was determined using Student’s *t* test (*, *P* < 0.05). Download FIG S3, PDF file, 0.8 MB. Copyright © 2017 Schuster et al. 2017 Schuster et al. This content is distributed under the terms of the Creative Commons Attribution 4.0 International license.

10.1128/mSphere.00333-17.4 FIG S4  Size profile of total, unmapped small RNAs obtained from control cells and RIG-I- and MDA5-deficient (KO) cells after infection for 24 h with SINV at an MOI of 0.1, for 48 h with YFV17D at an MOI of 0.1, or for 16 h with CBV3 at an MOI of 0.01. Download FIG S4, PDF file, 0.8 MB. Copyright © 2017 Schuster et al. 2017 Schuster et al. This content is distributed under the terms of the Creative Commons Attribution 4.0 International license.

Together, our results suggest that mammalian RNAi does not contribute to antiviral immunity in RIG-I- and MDA5-deficient cells, although we cannot rule out that other cell types or specific cellular niches support Dicer-dependent processing of viral long dsRNA into vsiRNAs. As it had been proposed previously that the interferon response masks or inhibits the RNAi-dependent processing of viral dsRNA (13), we analyzed viral small RNAs in wild-type virus infections of a clonal cell line deficient in both RIG-I and MDA5 sensors. We found no evidence for vsiRNA accumulation, suggesting that the interferon response is not sufficient to explain the lack of vsiRNA production in differentiated epithelial cells. Our study thus confirms and extends previous studies in differentiated cells (14, 15) by suggesting that RIG-I and MDA5 do not compete with Dicer-dependent processing of viral dsRNA. Our results fit the observation that human Dicer lacks the processivity required for efficient vsiRNA production (10), unlike the truncated Dicer variant lacking the N-terminal helicase domain found in mouse oocytes (25).

The IFN and RNAi responses coexist in somatic cells; however, Maillard et al. suggested that a functional IFN pathway masks or inhibits the activity of RNAi (13). These authors showed that differentiated mouse embryonic fibroblasts (MEFs) deficient in the signaling molecule MAVS or the IFN-α/β receptor (IFNAR1) were able to process exogenously provided dsRNA for sequence-specific gene silencing. In accordance, ∼22-nt siRNAs could be detected by Northern blot analysis in IFNAR1-deficient cells upon dsRNA transfection. However, it remained to be established whether dsRNA of viral origin would likewise be processed in the absence of an IFN response. Our study now provides evidence that the absence of an interferon response is not sufficient to uncover vsiRNA production, suggesting that other features regulate antiviral RNAi in mammals. In line with our observations, Argonaute 2 inactivation in MAVS-deficient cells did not result in consistent increases in replication of several RNA viruses, including influenza A virus (IAV) lacking its interferon/RNAi suppressor protein NS1 (IAV ΔNS1) (13). Yet, the latter observation is at odds with a recent study that identified AGO2-associated vsiRNAs upon infection with IAV ΔNS1 and observed RNAi-dependent restriction of viral replication (8). The basis for the discrepancy between these studies remains to be resolved.

Although our data suggest that RNAi does not have antiviral activity in differentiated IFN-deficient cells, it has been proposed that RNAi has antiviral activity in stem cells, which are also less responsive to IFN. For example, it was shown that (nonviral) dsRNA is processed into siRNAs in mESCs, mouse embryonic teratocarcinoma cell lines, and oocytes (26–28). Moreover, EMCV infection of mESCs resulted in Dicer-dependent accumulation of ∼22-nt vsiRNAs that are incorporated into AGO2, which was lost upon differentiation (9). The IFN response pathway is attenuated in mESCs (18, 19), and it therefore needs to be established whether the lack of the interferon response or other stem cell characteristics contribute to the antiviral activity of RNAi in those cells.

One aspect that should be taken into consideration is the choice of viruses used in these studies. Recent studies have suggested that inactivation of VSR activity in a virus uncovers antiviral RNAi in mammalian cells, including differentiated somatic cells (7, 8, 17). In contrast to previous work in which VSR-deficient viruses were used (7, 8, 13), we have chosen to study wild-type viruses to establish whether inactivation of the interferon response is sufficient to uncover vsiRNAs. To date, no suppressors of RNAi have been identified for SINV or CBV3, two of the viruses used in this paper. A recent report proposed that capsid protein of YFV binds dsRNA to suppress RNAi in mosquitoes, although this had not yet been demonstrated in an authentic YFV infection (23). We do note, however, that in plants and insects vsiRNAs are readily detected in infections with viruses that are known to encode VSRs (2), indicating that a sufficiently potent antiviral RNAi response would produce vsiRNAs even in the presence of VSRs.

Although our results suggest that cytosolic RNA sensors do not compete with Dicer for dsRNA, complex interactions between small RNA pathways and the interferon response seem to exist. For example, activation of the IFN response by viral infection led to the inactivation of AGO2 and derepression of miRNA-mediated repression of ISGs (29). The ongoing dissection of small RNA pathways in mammals will undoubtedly uncover more complexities and lead to a better understanding of their contribution to antiviral immunity.

### CRISPR.

Guide RNAs were designed using the online tool from the Zhang laboratory at the Massachusetts Institute of Technology (MIT) (http://crispr.mit.edu/) (30). The following guides were used: for RIG-I, guide 57 F, 5′-AAACAACAAGGGCCCAATGG-3′; guide 57 R, 5′-CCATTGGGCCCTTGTTGTTT-3′; guide 59 F, 5′-CTAGGGCATCCAAAAAGCCA-3′; and guide 59 R, 5′-TGGCTTTTTGGATGCCCTAG-3′; and for MDA5, guide 79 F, 5′-CGAATTCCCGAGTCCAACCA-3′; guide 79 R, 5′-TGGTTGGACTCGGGAATTCG-3′; guide 83 F, 5′-CGTCTTGGATAAGTGCATGG-3′; and guide 83 R, 5′-CCATGCACTTATCCAAGACG-3′. A 100 µM concentration of the forward and reverse oligonucleotides was phosphorylated using T4 polynucleotide kinase (PNK; Roche) for 1 h at 37°C. The mixture was incubated at 95°C for 5 min and slowly cooled to room temperature. The pCRISPR-hCAS9-1xgRNA-Puro plasmid was obtained from Martijn Langereis (31), digested with SapI, and treated with calf intestinal alkaline phosphatase (CIP; Roche). The guides were cloned into the vector and transformed into competent bacteria. Sanger sequencing was used to validate the incorporation of the correct guide. HeLa cells were seeded at a concentration of 1.5 × 10^5^ cells/ml in 6-well plates in a volume of 2 ml of Dulbecco’s modified Eagle’s medium (DMEM) supplemented with 10% fetal calf serum (FCS; Gibco) and penicillin-streptomycin (Gibco). Cells were transfected with 2.5 µg/well CRISPR-hCAS9-1xgRNA-Puro plasmids in total (cotransfection of two guide RNAs) using 7.5 µl/well FuGENE 6 (Promega). One day after transfection, 2 μg/ml puromycin was added to the cells for 3 days. Surviving cells were seeded in single-cell dilutions, cultured, and expanded into flasks. Genomic DNA was isolated from individual clones, and the presence of a deletion in the MDA5 gene was analyzed on an agarose gel after a diagnostic PCR using primers flanking the expected target sites (described below). In the clones carrying the expected 192-nt deletion in MDA5, a deletion in the RIG-I gene was introduced by repeating the transfection and selection procedure using two guide RNAs. The deletions in RIG-I and MDA5 were validated by Sanger sequencing.

**DNA sequencing.** Genomic DNA of CRISPR-Cas9-edited cells was isolated using the Quick-gDNA MiniPrep kit (Zymo Research). The genomic regions flanking the targeted sites in RIG-I and MDA5 were PCR amplified and sequenced using the primers RIG-I F (5′-CCAATGCTGTGACTTGGTAC-3′), RIG-I R (5′-TGTCTCAGACTAAGAGGCAT-3′), MDA5 F (5′-AGACCCTGCTTCTCTAAGTG-3′), and MDA5 R (5′-GCCTGCTTTGCAAAATCTGC-3′).

### Cell culture and poly(I⋅C) transfection.

HeLa cells were cultured in Dulbecco’s modified Eagle’s medium (DMEM) with 4.5 g/liter d-glucose (Gibco), 50 U/ml penicillin, 50 μg/ml streptomycin (Gibco), and 10% FCS (Gibco) in 5% CO_2_ at 37°C. For poly(I⋅C) transfections, cells were seeded at a density of 7.5 × 10^4^ cells in 500 µl in a 24-well plate. The next day, the cells were stimulated with 200 ng/well poly(I⋅C) (GE Healthcare) for 24 h using 0.3 µl/well FuGENE 6 (Promega).

### RT-qPCR.

RNA was isolated from poly(I⋅C)-stimulated samples using RNA-Solv reagent (Omega Biotek) or the NucleoSpin 96 RNA Core kit (Macherey-Nagel) according to the manufacturers’ protocols. RNA was treated with DNase I, and cDNA was generated using TaqMan reverse transcription reagents with random hexamers (Thermo Fisher) according to the manufacturer’s instructions. Gene expression was measured by RT-qPCR using the GoTaq qPCR master mix (Promega) and primers IFN-β F (5′-GCTTGGATTCCTACAAAGAAGCA-3′), IFN-β R (5′-ATAGATGGTCAATGCGGCGTC-3′), ISG15 F (5′-AAGGCGCAGATCACCCAGAAGAT), ISG15 R (5′-TCAGAGGTTCGTCGCATTTGTCCA), OAS1 F (5′-TTCCTGAAGGAAAGGTGCTTCCGA), OAS1 R (5′-AAGACAACCAGGTCAGCGTCAGAT), actin F (5′-CCTTCCTGGGCATGGAGTCCTG-3′), and actin R (5′-GGAGCAATGATCTTGATCTTC-3′). Experiments were analyzed using the threshold cycle (ΔΔ*CT*) method (32).

### SDS-PAGE and Western blotting.

HeLa control cells and RIG-I–MDA5-deficient cells were seeded in 6-well plates at a density of 1.5 × 10^5^ cells/ml in 2 ml of medium. Cells were stimulated with 200 ng/well poly(I⋅C) for 24 h using FuGENE 6 (Promega). Cells were collected in lysis buffer (10 mM Tris-HCl [pH 7.5], 150 mM NaCl, 0.5 mM EDTA, 2.5 mM MgCl_2_, 0.1% SDS, 1% Triton, 1% sodium deoxycholate), rotated at 4°C for 30 min, and centrifuged for 30 min at 15,600 × *g*. Primary antibodies anti-MDA5 (33), anti-RIG-I (ProSci), and antitubulin (Sanbio) were diluted 1:10,000, 1:500, and 1:1,000, respectively, in phosphate-buffered saline (PBS) containing 0.1% (vol/vol) Tween and 2.5% (wt/vol) nonfat dry milk. Secondary antibodies IRDye anti-rabbit (Li-Cor Biosciences) and IRDye anti-mouse (Li-Cor Biosciences) were diluted 1:5,000 in PBS containing 0.1% (vol/vol) Tween, 2.5% (wt/vol) nonfat dry milk, and 0.01% (wt/vol) SDS.

### Preparation of small RNA libraries.

 HeLa control cells or RIG-I- and MDA5-deficient cells were seeded at a density of 1.5 × 10^5^/ml in 2 ml of medium in two wells of a 6-well plate. Cells were infected with Sindbis virus for 24 h at a multiplicity of infection (MOI) of 0.1, yellow fever virus 17D strain for 48 h at an MOI of 0.1, and coxsackievirus B3 for 16 h at an MOI of 0.01. Samples were harvested in RNA-Solv reagent (Omega Biotek). Total RNA was isolated and separated by electrophoresis on a 15% acrylamide-bisacrylamide, 7 M urea, 0.5× Tris-borate-EDTA (TBE) gel flanked by radioactive RNA size markers of 19 nt, 24 nt, and 33 nt. The 19- to 30-nt fraction of each sample was excised and used to prepare small RNA libraries using the Illumina small RNA TruSeq kit according to the manufacturer’s instructions. The libraries were sequenced on an Illumina HiSeq 4000 system as 50-nt single-end reads according to Illumina’s instructions. The following quality controls were performed: base quality (Q30), base sequence content, duplicated reads, and FastQScreen. Small RNA sequences were analyzed using the Galaxy ToolShed at https://usegalaxy.org (34). The Bowtie for Illumina version 1.1.2 tool was used for mapping against the following genomes and miRNAs: SINV pTE3′2J (35), YFV17D (GenBank accession no. X03700.1), CBV3 (GenBank accession no. JX312064.1), and *Homo sapiens* (GRCh38) miRNA stem-loops, retrieved on 20 June 2017 from miRBase (36), allowing 1 mismatch in the first 28 nucleotides.

### Accession number(s).

Small RNA sequence data have been deposited in the NCBI Sequence Read Archive under BioProject accession number PRJNA397310.
