# Supplementary material for: Deletion of Cytoplasmic Double-Stranded RNA Sensors Does Not Uncover Viral Small Interfering RNA Production in Human Cells
Source: mSphere. 2017 Aug 16;2(4):e00333-17. doi: 10.1128/mSphere.00333-17 (PMC5557678; doi:10.1128/mSphere.00333-17)
Supplement: TEXT S1 [file sph004172342s5.pdf]

## Supplemental text

To assess whether our model viruses inhibit RNAi in mammalian cells, we established a luciferase-based RNAi reporter assay in which expression of firefly luciferase is silencing by a short hairpin RNA (shRNA). Processing of the shRNA into a functional siRNA is Dicer dependent and its silencing activity requires Argonaute 2 (Fig. S1A). Using this assay, we found that efficiency of RNAi in HeLa cells infected with Sindbis virus, yellow fever virus 17D, and coxsackievirus B3 was similar to RNAi efficiency in mock infected HeLa cells (Fig. S1B). These results suggest that these viruses do not suppress RNAi under our experimental conditions.

To extend these observations, we used a well-established assay in *Drosophila* S2 cells to identify viral suppressors of RNAi (1-3). Also under these conditions, we were unable to detect RNAi-suppressive activity of Sindbis virus (Fig. S1C). Published observations also suggest that Sindbis virus does not block Dicer function. For example, viral siRNAs are readily detectable in *Aedes aegypti* mosquitoes infected with Sindbis virus, but strongly reduced in infections with a Sindbis recombinant virus expressing the B2 protein of Flock House virus, a well-established dsRNA-binding RNAi suppressor that inhibits Dicer processing (4). Likewise, a direct biochemical assay for Dicer-2 function in lysates of the Aag2 cell line (derived from *Aedes aegypti* mosquitoes) indicates that dsRNA is efficiently processed into siRNAs in cells infected with a Sindbis recombinant expressing GFP, but not in cells infected with Sindbis recombinant expressing B2 (5).

## Supplemental Materials and methods

***RNAi reporter assay in Drosophila cells.*** Recombinant Sindbis virus expressing the Blasticidin resistance gene under control of a second subgenomic promoter was produced as described previously (1). The gene encoding Blasticidin resistance was transferred from the pcDNA6 vector (Invitrogen) into pTE3'2J vector (6). The resulting plasmid was linearized, in

vitro transcribed, and the RNA was transfected into BHK-21 cells to produce recombinant Sindbis virus, as described (1). *Drosophila* S2 cells (Invitrogen) were infected with the virus, infected cells were recovered under selection of a lethal concentration of blasticidin (Thermofisher) and subjected to a luciferase-based RNAi assay as described previously (1-3).

***RNAi reporter assay in mammalian cells.*** *Renilla reniformis* and firefly (Photinus pyralis) luciferase were expressed from pRL-TK (Promega) and pGL3-TK. pGL3-TK was generated by replacing the Renilla sequence in pRL-TK with the luciferase (GL3) sequence from pGL3 control (Promega). U6 promoter-driven short hairpin RNA expression cassettes were produced using a PCR-based approach (7) and cloned into pCR2.1 (Invitrogen). The specific shRNA plasmid targets the sequence GGTGGACATCACTTACGCTGA. The control shRNA plasmid specifically targets an earlier generation luciferase construct (GL2), but is inactive against pGL3-TK due to the presence of three mismatches.

Mouse embryonic fibroblasts or HeLa R19 cells were cotransfected with shRNA plasmid, pRL-TK and pGL3-TK using Lipofectamin 2000 (MEFs, Thermofisher) or FuGENE 6 (HeLa cells, Promega). Respectively, three or two days post-transfection, cell lysates were assayed for luciferase expression using the Dual-Luciferase Reporter Assay System (Promega) and a Tecan Ultra-evolution platereader or a Turner BioSystems luminometer. Fibroblasts from wildtype and Argonaute 2 (Ago2) knock-out mouse embryos were kindly provided by G. Hannon (Cold Spring Harbor Laboratories) and cultured in Dulbecco's modified medium (DMEM) supplemented with 10% fetal calf serum, 1 mM sodium pyruvate and 50 U/mL penicillin and 50 μg/mL streptomycin (7).

For the RNAi reporter assay in mock or virus-infected cells, HeLa R19 were cultured in DMEM supplemented with 10% fetal calf serum and 50 U/mL penicillin and 50 μg/mL streptomycin and seeded at a density of $1x10^4$ cells per well in a 96-well format. The same day, plasmids encoding shRNAs (luciferase or non-targeting control) along with TK-Ren and

TK-Luc were co-transfected using FuGENE 6 transfection reagent (Promega). Transfected cells were mock-infected for 24 h, or infected with Sindbis virus for 24 h at an MOI of 0.1, Yellow fever virus 17D strain for 46 h at an MOI of 0.1, or Coxsackievirus B3 for 16 h at an MOI of 0.01. Cells were lysed 48 h after transfection. Statistical significance was determined using a Student's t-test (*, $P < 0.05$, ns = non-significant).

## Supplemental references


1. **van Mierlo JT, Overheul GJ, Obadia B, van Cleef KW, Webster CL, Saleh MC, Obbard DJ, van Rij RP.** 2014. Novel Drosophila viruses encode host-specific suppressors of RNAi. PLoS Pathog **10:**e1004256.
2. **van Rij RP, Saleh MC, Berry B, Foo C, Houk A, Antoniewski C, Andino R.** 2006. The RNA silencing endonuclease Argonaute 2 mediates specific antiviral immunity in Drosophila melanogaster. Genes Dev **20:**2985-2995.
3. **van Cleef KW, van Mierlo JT, van den Beek M, van Rij RP.** 2011. Identification of viral suppressors of RNAi by a reporter assay in Drosophila S2 cell culture. Methods Mol Biol **721:**201-213.
4. **Myles KM, Wiley MR, Morazzani EM, Adelman ZN.** 2008. Alphavirus-derived small RNAs modulate pathogenesis in disease vector mosquitoes. Proc Natl Acad Sci U S A **105:**19938-19943.
5. **Castanotto D, Li H, Rossi JJ.** 2002. Functional siRNA expression from transfected PCR products. RNA **8:**1454-1460.
6. **Hahn CS, Hahn YS, Braciale TJ, Rice CM.** 1992. Infectious Sindbis virus transient expression vectors for studying antigen processing and presentation. Proc Natl Acad Sci U S A **89:**2679-2683.
7. **Liu J, Carmell MA, Rivas FV, Marsden CG, Thomson JM, Song JJ, Hammond SM, Joshua-Tor L, Hannon GJ.** 2004. Argonaute2 is the catalytic engine of mammalian RNAi. Science **305:**1437-1441.
