# Supplementary figures and images for: Deletion of Cytoplasmic Double-Stranded RNA Sensors Does Not Uncover Viral Small Interfering RNA Production in Human Cells
Source: mSphere. 2017 Aug 16;2(4):e00333-17. doi: 10.1128/mSphere.00333-17 (PMC5557678; doi:10.1128/mSphere.00333-17)

**A**

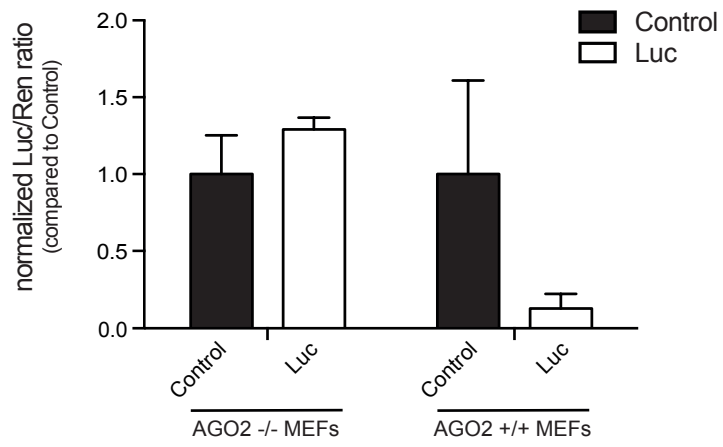

**B**

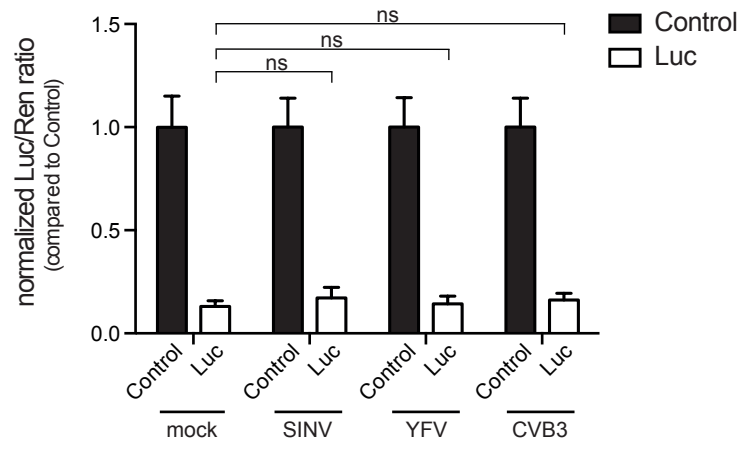

**C**

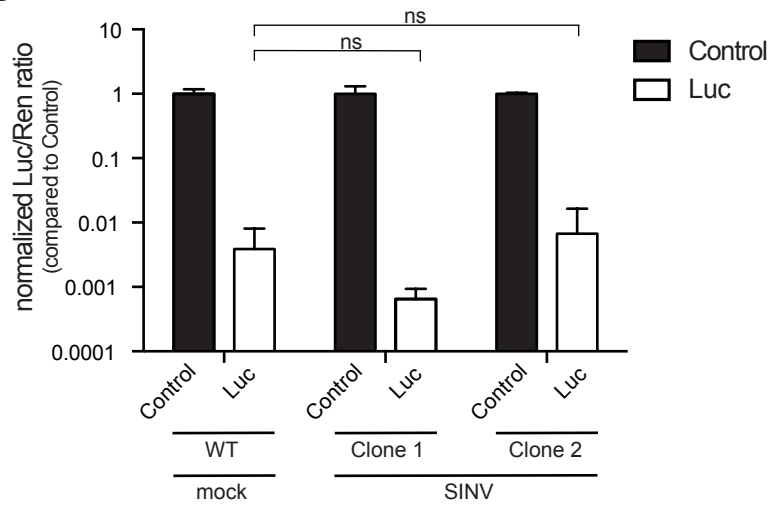

Supplement: FIG S1 [file sph004172342sf1.pdf]

RIG-I

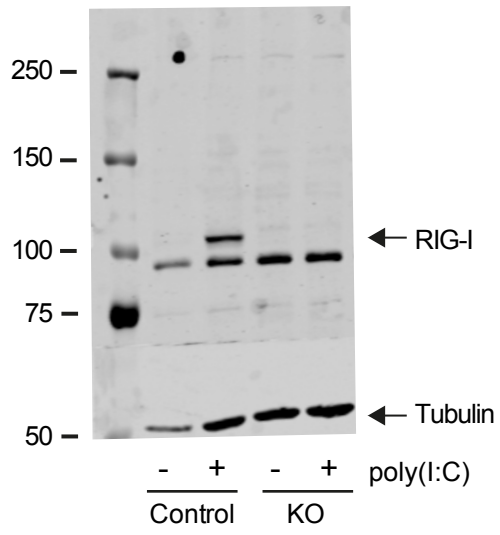

MDA5

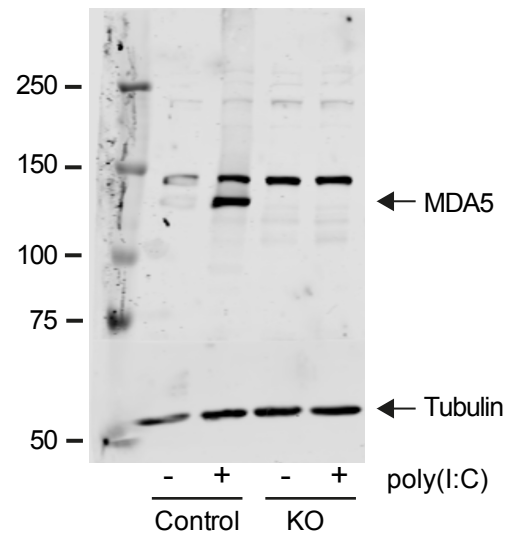

Supplement: FIG S2 [file sph004172342sf2.pdf]

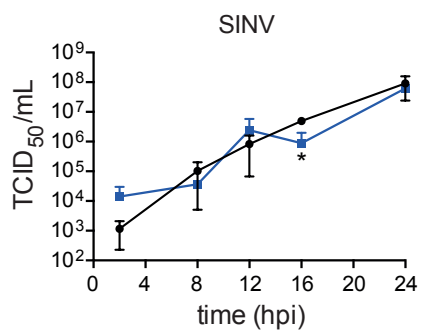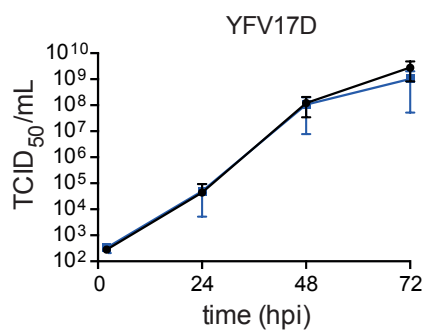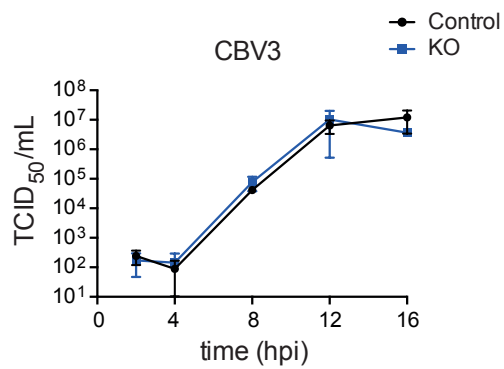

Supplement: FIG S3 [file sph004172342sf3.pdf]

SINV

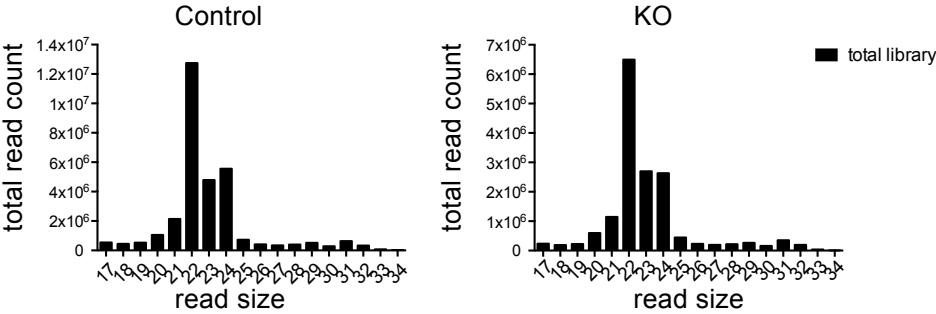

YFV17D

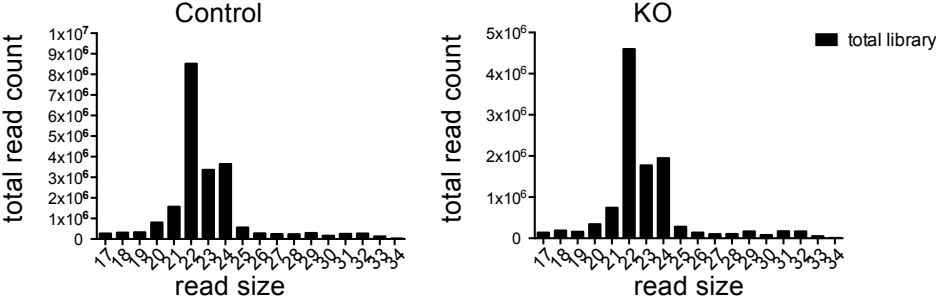

CBV3

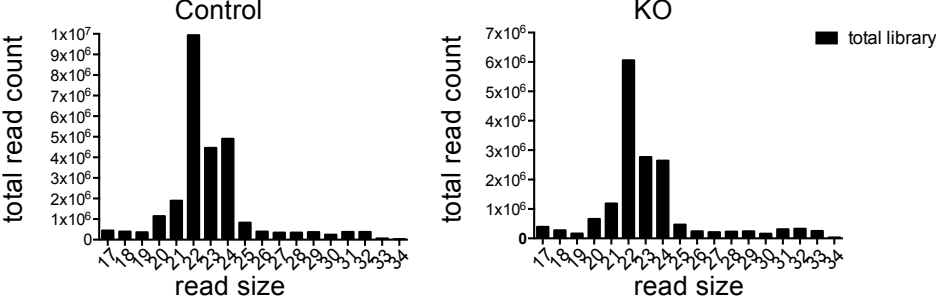

Supplement: FIG S4 [file sph004172342sf4.pdf]
